# Kobra Surgery Simulator—A Possibility to Improve Digital Teaching? A Case-Control Study

**DOI:** 10.3390/ijerph18041827

**Published:** 2021-02-13

**Authors:** Mayte Buchbender, Mathias Maser, Friedrich W. Neukam, Marco R. Kesting, Sameh Attia, Christian M. Schmitt

**Affiliations:** 1 Department of Oral and Maxillofacial Surgery, University of Erlangen-Nuremberg, Glückstraße 11, 91054 Erlangen, Germany; mathias-maser@gmx.de (M.M.); Friedrich.Neukam.extern@uk-erlangen.de (F.W.N.); Marco.Kesting@uk-erlangen.de (M.R.K.); schmitcn@outlook.de (C.M.S.); 2 Department of Oral and Maxillofacial Surgery, University of Giessen, 35392 Giessen, Germany; sameh.attia@dentist.med.uni-giessen.de

**Keywords:** coronavirus, oral surgery, COVID-19, surgical skills, surgery simulator

## Abstract

Computer-aided simulations have long been of great importance in university teaching; however, to date, there is limited use of such simulations in the dental surgical sector. For this purpose, an oral surgery simulator, “Kobra”, was implemented in student training and was evaluated for dental education. Dental students (group 1, third-year and group 2, fourth-year) and dentists of the faculty (control group) were trained to use the simulator. The outcomes for group 1 (apicoectomy of an upper lateral incisor with Kobra), group 2 (removal of an impacted lower wisdom tooth with Kobra) and the control group (both procedures with Kobra) were evaluated. For evaluation purposes, subjective parameters (improvement of practical skills, comparison between conventional training and Kobra simulation, and implementation of simulation-based teaching) and objective parameters (removal of bone, tooth substance and soft tissue measured while performing the Kobra simulation) were assessed using questionnaires with a scale ranging from 1–5. A total of 49 students (third-year *n* = 29, with 22 women and 7 men; fourth-year *n* = 20, with 17 women and 3 men) and 10 dentists (women *n* = 5 and men *n* = 5) participated. Compared to the Kobra simulation, the conventional training method with plastic models was still favored (the difference was non-significant). Compared to the dentists, the simulation data showed a less precise surgical performance of the students (the difference was not significant). The Kobra simulation may offer an additional method to conventional surgery training using plastic models, with benefits for students and faculty staff.

## 1. Introduction

The changing trend towards digitalization is not new territory in dentistry. With increased possibilities for digital impressions to be taken using different intraoral scanners as well as the digital planning and creation of prosthetics for patients (using computer-aided design (CAD)/computer-aided manufacturing (CAM)), dentists are able to treat patients in a digital way. In addition, in the field of dental education, there are more possibilities to digitalize. For this purpose, simulation systems have been tested within the framework of medical and dental training [1,2,3].

Digital computer-aided teaching can both improve cognitive abilities and have a positive effect on sensorimotor skills. Participants who played video games for at least three hours per week scored significantly better in simulating a robot suturing exercise [4,5]. A study by Aliaga et al. [1] compared the conventional processing of a methacrylate block to a corresponding simulator, with students at the same level of knowledge; these two teaching methods were considered equivalent. In another study, cavities on plastic teeth were prepared conventionally and with the aid of a simulator; the latter method led to significantly better results [2]. A comparable study with the same simulator evaluated first-year and fifth-year students and already licensed dentists and showed that the simulator was a very suitable teaching module for practical training [2]. Until recently, the usual practical student course for surgical training took place on plastic models; teeth were extracted by osteotomy and surgical procedures such as apicoectomies were simulated [6]. A surgical digital simulation, as an added value to previous methods, has not been mentioned in the literature thus far. The rapid shift in thinking toward digitalization will affect universities by 2020 at the latest.

The novel coronavirus, which was classified as a pandemic by the World Health Organization (WHO) in 2020 [7,8], has changed and affected structures within the economy and politics as well as impacted the life of every individual [9,10,11]. The novel situation that has arisen as a result of the coronavirus has had an impact not only on the clinical everyday life of every dentist and physician but also on the everyday teaching routines at university hospitals [12,13,14,15,16,17,18,19]. Because of the increased risk of becoming infected with the coronavirus (COVID-19) among dental staff due to the possible formation of aerosols during rotating work on teeth or in the patient’s mouth, the practically oriented student education using patients cannot take place as usual [18,19,20,21,22]. The WHO has recommended measures such as triaging patients upon entering university clinics or only performing emergency treatment on patients [23,24]. The usual classroom teaching in large group cohorts for theoretical and practical training had to be stopped due to strict hygiene regulations [25]. Only block internships in small groups of up to four students could take place, in compliance with the distance regulations and using appropriate protective clothing [26]. Because of the rotating instruments under a cooling medium, a certain aerosol formation also occurs in this situation, even if no real patient is involved [21,25,26]. The distance regulation between two students can only partially be observed here, since the usual treatment requires an assistant to perform water suction [26,27]. As a result of these requirements, and in order to contain the virus, new approaches are required more than ever in the practical training of future dentists [28].

Apart from the coronavirus, since 2017 students of the Department of Oral and Maxillofacial Surgery of the University Erlangen-Nuremberg have been able to perform tooth extractions, osteotomies, and apicoectomies on a simulator (Kobra Simulator, Forsslund systems AB, Stockholm, Sweden). However, the reported data about simulators for dental surgical education are lacking. Virtual training should not only benefit students, but faculty members must also be able to consider its input as positive. Thus, we hypothesize that dental students will perform more accurate simulation surgery, compared to dentists of the faculty, when using the simulator. In this case-control study, we aimed to investigate how dentists and students perform in the Kobra simulation, using objective and subjective parameters in order to consider a possible effect of the simulator or its use to improve oral surgical skills in further teaching.

## 2. Experimental Section

### 2.1. Study Design and Setup

This study reports a case-control data analysis of students and dentists within the Friedrich-Alexander University of Erlangen-Nuremberg (FAU). The students included in this study participated in a surgery course within the Department of Oral and Maxillofacial Surgery, University of Erlangen-Nuremberg, from April 2017 to July 2017. The following PECO design was used: P (population): member of a dental university; E (exposure): dental students; C (comparator): dentists; O (outcome): amount of removed parameter within the virtual operation on the simulator.

We included participants of both genders and all ages after obtaining signed, informed consent. Two groups were distinguished according to the expected and taught theoretical knowledge level and examined in comparison to the control group. The first group was students in their third year, the second group was students in their fourth year, and the control group consisted of licensed dentists within the dental clinic of FAU. The groups were chosen from different years of study, as they had different levels of surgical experience. The control group was formed from dentists who graduated from the same faculty and had similar surgical skills or experience. Dentists with more or less experience or other types of surgical training during their studies were excluded.

Because of the design of the simulator, left-handed individuals were had also excluded.

Using the simulator, the participants of group 1 performed an apicoectomy of an upper front tooth, the students in group 2 performed an extraction of a wisdom tooth in the lower jaw, and the control group performed both surgeries. Groups 1 and 2 also did standard exercises on plastic models (apicoectomy, extraction of teeth, and removal of wisdom teeth in the lower jaw) within the surgical course content of the department, independently from the Kobra simulation.

All groups were briefed equally about the theory of apicoectomy or the extraction of a wisdom tooth and had ten minutes to practice with the simulator before performing the surgical procedures. In order to limit the time of the exercise and to make sure that each participant had the same amount of time during the practice and the exercise, the time was limited to 10 min. This is the time required by one oral surgeon from the department for each of the exercises. The time during the surgery and practice was measured with a stopwatch.

After the virtual operation, the removed parameters during the simulation were collected, and each participant received an evaluation sheet according to the assigned group. In addition to the specific questions about the simulator, which are described in Section 2.5, the non-specific section asked about age, gender, clinical experience, and video gaming.

### 2.2. Design of the Simulator

**Figure 1:**
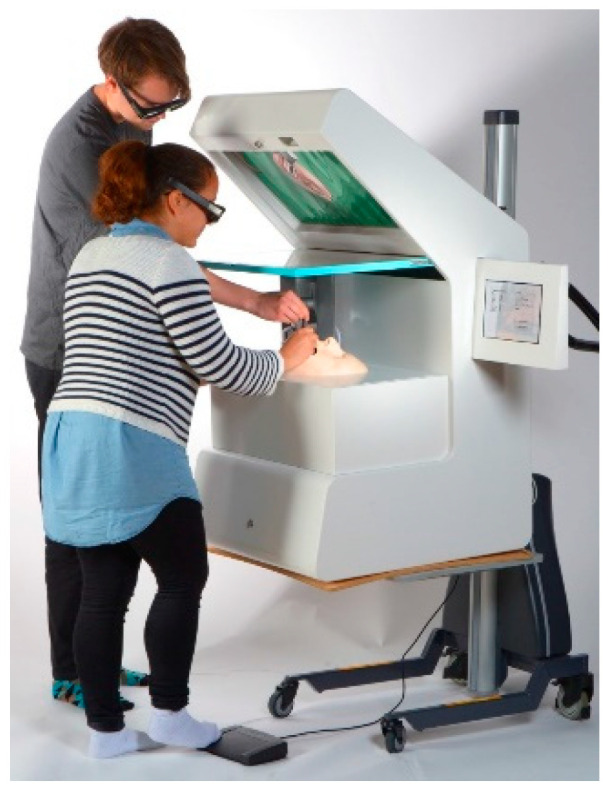
The structure of the Kobra simulator during virtual surgery.

**Figure 2:**
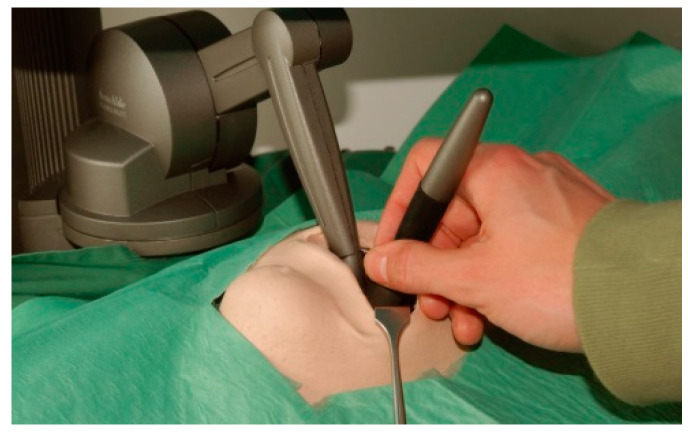
The haptic device of the Kobra simulator as used in virtual surgery.

The Kobra simulator as used in the simulation of an oral surgical procedure is shown in Figure 1. This simulator consists of a phantom head, a 3D screen, a tablet for selecting the patient case insight of relevant virtual patient data (i.e., X-rays), two 3D glasses (Nvidia 3D and Vision 2), a foot control for the handling of the surgical handpiece and an advanced joystick with touch feedback, a so-called haptic device (Figure 2). Depending on the virtually represented surface, such as bone, enamel or dentin, the simulated resistance to abrasion varies. Only the instrument tip is included in the simulation. The performance of anesthesia, flap design, wound closure or any other supportive measurements after surgical intervention is not part of the simulation.

### 2.3. Simulation of Apicoectomy on an Upper Anterior Tooth

The short anamnesis of the virtual patient is presented as follows. A 38-year-old female patient needs medical attention because of pain in tooth 22, which is sensitive to bite. Clinically the tooth is not loosened, is slightly sensitive to percussion and has been treated with a sufficient root filling. According to the corresponding X-ray, the treatment plan is an apicoectomy on tooth 22.

**Figure 3:**
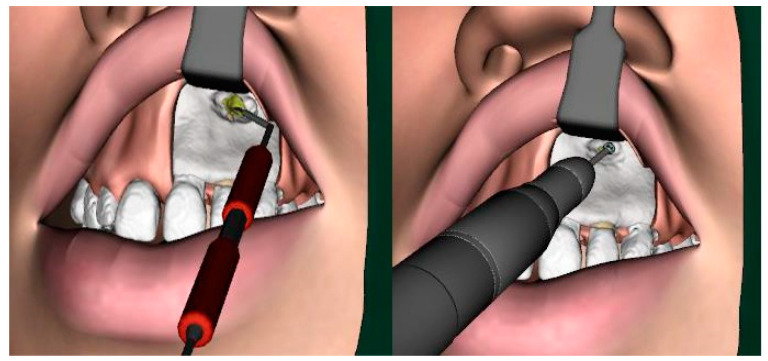
The virtual intraoperative situation while drilling the root tip cavity and removing infected (yellow) tissue with a surgical spoon.

Figure 3 shows the operating site of the simulation and the removal of bone with the rotating handpiece and of infected tissue around the root tip of the tooth with the surgical spoon.

### 2.4. Simulation of Wisdom Tooth Extraction

The short anamnesis of the virtual patient is presented as follows. The patient is a 28-year-old man who requires surgical removal of a wisdom tooth. The aim of this exercise is the complete and safe extraction of the wisdom tooth. Participants must examine the radiograph and plan the extraction accordingly.

**Figure 4:**
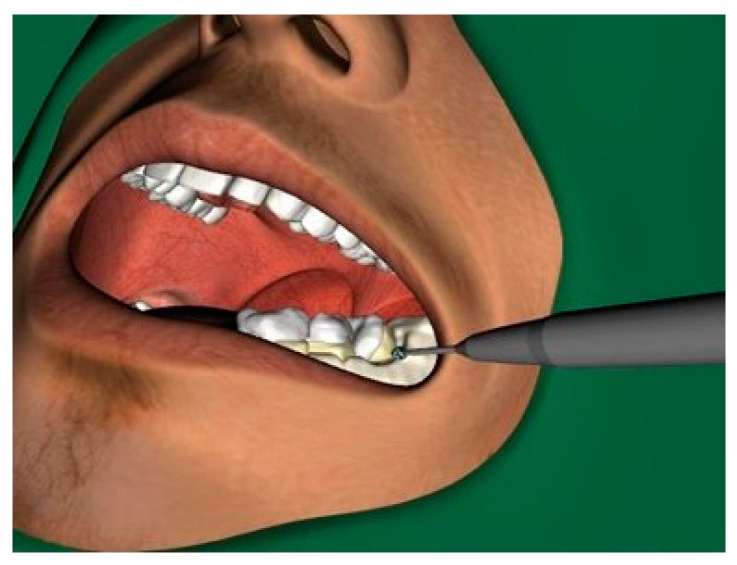
The virtual intraoperative situation while drilling to remove a wisdom tooth in the left lower jaw.

Figure 4 shows the operating site of the simulated removal of bone and tooth structure to remove the wisdom tooth.

### 2.5. Data Extraction and Examination

We analyzed the following objective data during the simulation:

- Bone removal (in mm^3^)
- Removal of pulp (in mm^3^)
- Removal of dentine (in mm^3^)
- Removal of enamel (in mm^3^)
- Removal of gutta-percha (in mm^3^)
- Removal of infected tissue (in mm^3^)

Removal of the substance of tooth or adjacent tooth (in mm^3^)

We analyzed the following data after simulation according to the evaluation sheet on a scale from 1 to 5, as follows: 1––not at all; 2––disagree in part; 3––neutral; 4––agree in part; 5––fully agree.

- Question 1: Whether simulation with the Kobra simulator should be an integral part of training in the future.
- Question 2: Whether there should be an implementation of new simulation cases.
- Question 3: Whether the Kobra simulation is haptically realistic.
- Question 4: Whether the Kobra simulation is a realistic simulation concerning the case presentation and the position of the surgical operating situs.
- Question 5: Which teaching method (plastic model or Kobra) would be preferred.

For the comparability of the parameters, they were objectified; this was done by removing the individual parameters on the simulator and the statements on the questionnaire and evaluating them using the previously defined scale (1–5) as described above. To keep the bias low, the plastic model exercises were not evaluated, and the comparability of the parameters was ensured as already described. The study size was not statistically determined in advance; those who wished to participate on a voluntary basis and after written consent were included.

### 2.6. Outcomes

Our primary outcome was defined as a comparison of the objective parameters during simulation between the two student groups and the control group. Our secondary outcome was a comparison of the subjective parameters.

### 2.7. Data and Statistical Analysis

Statistical analysis of the collected data was performed using the software SPSS, Version 25 (IBM, Armonk, NY, USA). For the statistical comparison of the objective data (bone removal, removal of pulp, removal of dentine, removal of enamel, removal of gutta-percha, removal of infected tissue, removal of the substance of a tooth or the adjacent tooth) between the two groups (either group 1 or 2 vs. control group), a non-parametric Mann-Whitney *U*-test with a significance level of *p* < 0.05 was applied. For the statistical comparison of the outcomes of questions 1–5 between the three groups, a non- parametric additional pairwise comparison with a Mann-Whitney *U*-test to detect statistical significance (significance level *p* < 0.05) was performed. Afterwards, the Bonferroni correction was performed, and the significance level was adapted to *p* < 0.0083 for objective parameters and *p* < 0.00625 for subjective parameters.

## 3. Results

### 3.1. Participant Cohort

A total of *n* = 59 (group 1, *n* = 29; group 2, *n* = 20; control group, *n* = 10) participated. Group 1 consisted of 22 female and seven male students, with an average age of 24.59 (±2.70) years.

Group 2 consisted of 17 female and three male students, with an average age of 24.40 (±3.15) years.

The gender distribution in the control group was five female dentists and five male dentists, with an average age of 28.22 (±2.73) years.

Within group 1, four students stated that they played video games regularly. Within group 2, no students stated that they played video games regularly. Among the control group, two dentists stated that they regularly played video games regularly.

Within group 1, 15 students had their own experiences with extractions in patients, whereas 14 participants had no surgical experience at all. Within group 2, 12 students had previously worked on a patient surgically, and eight students had not worked on a patient. Among the dentists, there was one person without any surgical experience and three others who had not performed wisdom tooth extraction. Only three dentists stated that they had performed an apicoectomy.

### 3.2. Primary Outcome—Objective Parameters

- Simulation of an apicoectomy of an upper front tooth (Group 1 vs. Control group)

**Figure 5:**
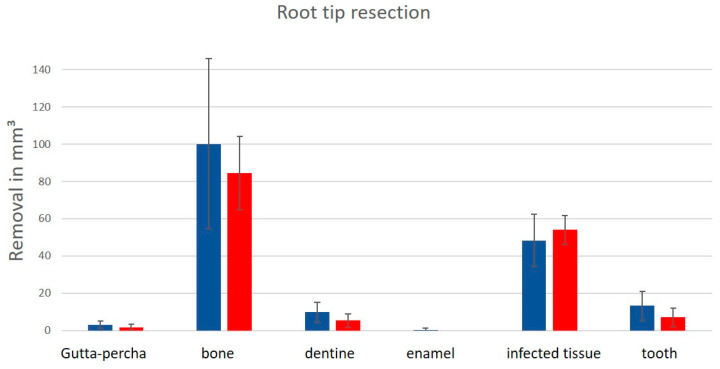
The removal of the parameters (gutta-percha, bone, dentine, enamel, infected tissue and tooth) during the virtual apicoectomy; blue: students; red: dentists. There was no significance between groups as determined by the Mann-Whitney *U*-test for *p* = 0.020 (gutta-percha); *p* = 0.010 (dentine); *p* = 0.009 (tooth) (*p* < 0.0083).

The different removals of the defined parameters by the Kobra simulator by the different groups (group 1 vs. control group) are illustrated in Figure 5. The Mann-Whitney *U*-test test revealed no significant differences (*p* < 0.0083) in both groups concerning the removal of gutta-percha (*p* = 0.020), dentine (*p* = 0.010) and tooth (*p* = 0.009). The students tended to have higher amounts of removal in the parameters, except the parameter of infected tissue, where dentists had a higher amount of removal.

- Simulation of wisdom tooth extraction (Group 2 vs. Control group)

**Figure 6:**
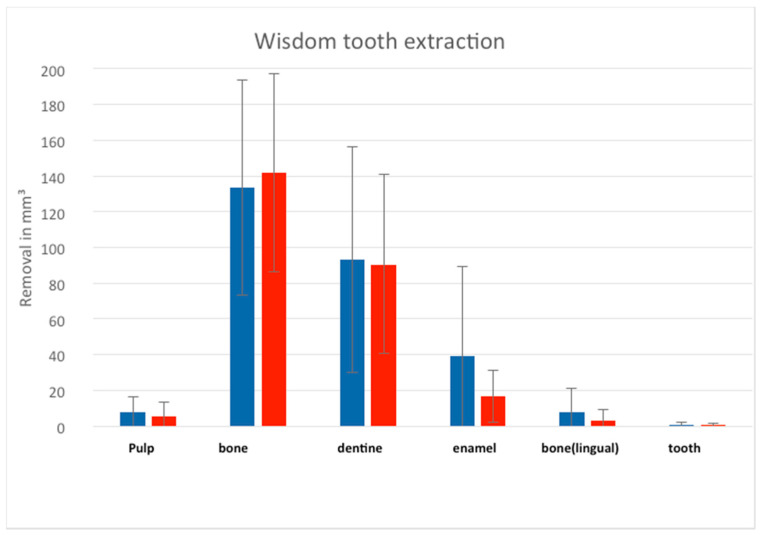
The removal of the parameters (pulp, bone, dentine, enamel, bone lingual and tooth) during the virtual removal of the wisdom tooth in the left lower mandible; blue: students; red: dentists, without any significances.

The differences between group 2 and the control group showed no significance according to the Mann-Whitney *U*-test (Figure 6). However, the median values between the two groups differed concerning the ablation of the pulp (group 1, 6.40 vs. group 2, 1.55) as well as the bone (128.7 vs. 124.75) and dentine (97.50 vs. 63.95).

### 3.3. Secondary Outcome—Subjective Parameters

Table 1 gives an overview of the evaluation of the subjective parameters using evaluation sheets. The question as to whether the simulator should be part of the training in the future (question 1) had no significance (*p* < 0.00625) between both groups (*p* = 0.012, group 2; *p* = 0.048, group 1). The control group scored this question with a mean of 4.22 and a maximum of 5. The question of whether new simulation cases should be integrated into the simulator in the future (question 2) showed a statistical significance, with *p* = 0.002 for group 1 and *p* = 0.005 for group 2. Questions 3 and 4 showed no statistically relevant differences, but the control group tended to rate these questions with a higher mean value.

Furthermore, it was determined which learning model (simulator vs. plastic model) was favored by the three groups (question 5). From group 1, four students were in favor of training alone through the simulator. Six wanted a combination of both (three each from groups 1 and 2). In the control group, there were five dentists in favor of sole training by the simulator and five favoring the plastic model.

## 4. Discussion

New technologies and ways to digitalize teaching methods were in demand even before the Coronavirus outbreak. Holding time-independent and location-independent teaching as digitally as possible would have advantages for students and also faculty members. However, the implementation of digital teaching methods was certainly advanced or forced in many places by the novel situation in 2020, since it posed new challenges to global society [8,9,11]. The life of every individual was restricted by hygiene regulations; furthermore, new teaching concepts were needed at every university [26]. Since dentists in particular belong to a high-risk group because of work-related aerosol generation [23,26], training students with the use of actual patients had to be suspended for several months [29]. In addition, practical surgical instruction in the usual small groups in the laboratory or on plastic models was not easily conducted during this time [30]. Hygiene concepts had to be developed quickly, but students also had to be taught constantly in practical terms [21,22,31].

Virtual simulation used to supplement apprenticeship thus quickly came to the attention of many [26]. From preliminary studies, we know that the use of a simulator can improve dental education [31,32,33,34]. However, previous studies on the simulator were limited to non-surgical areas. The exercises on the simulator involved the preparation of cavities in teeth or the processing of a methacrylate block. Especially among younger students in their earlier semesters, the use of simulators has led to major improvements in the course of their studies [1,2,3]. For younger students in particular, computer-assisted learning can be integrated into teaching as an integral and successful component through extended introduction time on the simulator and increased practice time. Compared to classroom teaching, participants experience more self-determination and develop more self-motivation [33]. It was also assumed that people who regularly play video games improve their skills and are therefore more successful in a simulation. In this study, however, only six participants reported playing video games regularly, so we did not consider a correlation between this observation and the virtual skills of the participants in the groups. However, the reported data about simulators for dental education are poor, because studies are few in number and above all lack comparison of comparable simulators [35]. For surgical simulation, only the Kobra simulator is available within the Department of Oral and Maxillofacial Surgery at FAU. In the subjective questioning of the three groups within this study, the control group (dentists) in particular was in favor of integrating the surgical simulator into future teaching. This is possibly due to the fact that the simulation offers teachers at university hospitals a considerable advantage in terms of time and the group management of students [36]. The students can practice on the simulation alone after instruction. By recording the parameters that have been removed, it is possible to go into more detail in a feedback discussion between the teacher and student, independent of time and location. This dialogue can even be held online via video call without special hygiene regulations, i.e., during a pandemic. Moreover, with the simulator, a supervision ratio situation of 1:1, and generally of 1:3, can thus be achieved much more easily. This was also made clear by this evaluation among advocates in the control group, with the significant implementation of new cases to the study groups (*p* = 0.002, group 1 and *p* = 0.005, group 2).

Regarding the objective parameters, we hypothesized that students would perform more accurately in the surgical simulation; this was not confirmed. There was a difference in the simulated apicoectomy between group 1 and the control group concerning the removal parameters of the different tissues (gutta-percha, *p* = 0.020; dentine, *p* = 0.010; tooth, *p* = 0.009), but this was not statistically significant. The students tended to have higher amounts of removal of these parameters. This finding may be explained by the fact that the younger students had not yet performed such operations themselves or had not seen them on real patients, while dentists can imagine or know in advance the optimal end result of such an apicoectomy. This exercise could be done with older students and compared to dentists to show a different effect. The degree of difficulty for an optimal operation is increased in this case, because for a decent apicoectomy, as little bone and dentine or tooth structure as possible, but as much gutta-percha from the orthograde filling and infected tissue as necessary, should be removed [37], which influences the outcome in the clinic.

There was no significance between group 2 and the control group regarding the removal of the tissues. In this group we had students of their fourth academic year, and according to previous surgical teaching, they had already followed this kind of simulated surgery on patients and performed it themselves. Thus, they could better visualize the outcome of the operation beforehand. Therefore, we can assume that the use of such a simulator paired with clinical experience could lead to a benefit for the students.

Moreover, due to the simulator’s ability to collect removal parameters during simulation interventions, it is able to assess each student’s performance objectively. The removal of parameters can be compared with an optimal operation by the university teacher and objectively graded on this basis. Here, too, it would be possible to generate an optimal coronavirus exam simulation without the need for a face-to-face exam for the entire semester. In addition, grading would be comprehensive and verifiable, which has advantages for both sides. In the course of the digitalization of teaching, new examination modalities can also be developed.

While the plastic model was favored by the study groups and also by half of the control group, it was considered a reasonable combination with the Kobra simulator.

Compared to real operations and the circumstances, the Kobra simulator has some drawbacks. Local anesthesia, incision and flap formation, and bleeding and wound closure are not part of the simulation. The simulation can be used for learning and understanding the actual surgical procedure without being distracted by bleeding or other accompanying events. It offers students the opportunity to gain surgical experience without patient contact. But the communication with the patient is missing. This soft skill should not be ignored, since in the daily life of a dentist, the patient relationship is built only on communication.

There are a few shortcomings in this study that need to be mentioned and critically discussed. The investigation modalities vary only slightly within the groups, so the results maybe heterogeneous. Although the level of knowledge in reality is not exactly the same for every participant, this influence reduces the possible heterogeneity. Moreover, it should be mentioned that the small number of participants as well as the similarity of many parameters and resultant analyses reduce the statistical power.

## 5. Conclusions

Dental students showed a larger amount of removal area during the virtual simulation and less precise surgical performance than dentists, especially in the group with younger students; between the older students and the dentists, no statistic difference was shown. Thus, computer-supported surgical simulation with the Kobra simulator cannot replace conventional training on the plastic model or on the patient. However, it may provide more clinically experienced students an additional digital teaching method and may also offer new opportunities for practical examination and benefit the faculty staff.

## Figures and Tables

**Table 1:** The mean values and median parameters (scale 1–5) of the subjective parameters of questions 1–4 with no significances in Mann Whitney *U*-test between the groups for *p* = 0.048; *p* = 0.012 in question 1; but a significance in question 2 * *p* = 0.002; ^#^ *p* = 0.005 (*p* < 0.00625).

	Group 1 (*n* = 29)	Group 2 (*n* = 20)	Control Group (*n* = 10)
	Mean Value (Range min.1–max.5)	Mean Value (Range min.1–max.5)	Mean Value (Range min.1–max.5)
Question 1	3.17	3.15	4.22
Question 2	3.38 *	3.55 ^#^	4.88
Question 3	3.28	3.55	4.00
Question 4	3.50	3.70	4.22

## Data Availability

The data presented in this study are available on request from the corresponding author.
